# Human Pancreatic Cancer Contains a Side Population Expressing Cancer Stem Cell-Associated and Prognostic Genes

**DOI:** 10.1371/journal.pone.0073968

**Published:** 2013-09-17

**Authors:** Anke Van den broeck, Hugo Vankelecom, Wouter Van Delm, Lies Gremeaux, Jasper Wouters, Joke Allemeersch, Olivier Govaere, Tania Roskams, Baki Topal

**Affiliations:** 1 Department of Abdominal Surgery, University Hospitals Leuven, Leuven, Belgium; 2 Laboratory of Tissue Plasticity, Research Unit of Embryo and Stem Cells, Department of Development & Regeneration, University of Leuven (KU Leuven), Leuven, Belgium; 3 VIB Nucleomics Core, University of Leuven (KU Leuven), Leuven, Belgium; 4 Department of Pathology, University Hospitals Leuven, Leuven, Belgium; Enzo Life Sciences, Inc., United States of America

## Abstract

In many types of cancers, a side population (SP) has been identified based on high efflux capacity, thereby enriching for chemoresistant cells as well as for candidate cancer stem cells (CSC). Here, we explored whether human pancreatic ductal adenocarcinoma (PDAC) contains a SP, and whether its gene expression profile is associated with chemoresistance, CSC and prognosis. After dispersion into single cells and incubation with Hoechst dye, we analyzed human PDAC resections specimens using flow cytometry (FACS). We identified a SP and main population (MP) in all human PDAC resection specimens (n = 52) analyzed, but detected immune (CD45^+^) and endothelial (CD31^+^) cells in this fraction together with tumor cells. The SP and MP cells, or more purified fractions depleted from CD31^+^/CD45^+^ cells (pSP and pMP), were sorted by FACS and subjected to whole-genome expression analysis. This revealed upregulation of genes associated with therapy resistance and of markers identified before in putative pancreatic CSC. pSP gene signatures of 32 or 10 up- or downregulated genes were developed and tested for discriminatory competence between pSP and pMP in different sets of PDAC samples. The prognostic value of the pSP genes was validated in a large independent series of PDAC patients (n = 78) using nCounter analysis of expression (in tumor *versus* surrounding pancreatic tissue) and Cox regression for disease-free and overall survival. Of these genes, expression levels of *ABCB1* and *CXCR4* were correlated with worse patient survival. Thus, our study for the first time demonstrates that human PDAC contains a SP. This tumor subpopulation may represent a valuable therapeutic target given its chemoresistance- and CSC-associated gene expression characteristics with potential prognostic value.

## Introduction

Despite large efforts to improve its prognosis, pancreatic cancer (pancreatic ductal adenocarcinoma or PDAC) remains a major cause of cancer-related mortality [1]. Delayed detection and therapy resistance are critical determinants of PDAC treatment failure. A better understanding of the mechanisms underlying therapy resistance is therefore essential. In several types of cancer, a side population (SP) has been identified as a subpopulation that enriches for cells that are chemo- and radioresistant due to the presence of multidrug transporters and the capacity to repair DNA damage and withstand apoptosis [2]. Based on their efflux capacity, SP cells are identified in flow-cytometric (FACS) analysis as a side branch of ‘Hoechst-low’ cells after incubation with the Hoechst33342 dye [3].

Therapy resistance is also considered a particular characteristic of so-called ‘cancer stem cells’ (CSC) [4]. CSC are thought to survive conventional therapies and therefore responsible for tumor relapse. Candidate pancreatic CSC populations have recently been identified based on cell membrane markers. CD24^+^/CD44^+^/epithelial cell adhesion molecule (EPCAM)^+^ cells and CD133^+^ cells were shown to display CSC properties [5], [6]. However, only partial overlap was found between these different pancreatic CSC populations, indicating the need for alternative identification strategies. In various cancer types, the SP has been shown to be enriched for CSC(-like) phenotype and activity [7]. Regarding pancreatic cancer, a SP was previously found in cultured cell lines [8]–[11], and recently, our group identified a SP in xenograft tumors grown from human PDAC samples in immunodeficient mice [12]. These SPs were demonstrated to possess chemoresistant capacity. However, it has not been shown whether PDAC directly from the patient, without intervening culture or expansion in the murine host environment, also contains a SP. In the present study, we report that PDAC isolated from patients harbors a SP and that this SP expresses genes associated with pancreatic CSC and chemoresistance, as well as with patient prognosis.

## Materials and Methods

### PDAC Samples and SP Analysis

Between 2008 and 2010, PDAC surgical resection specimens were obtained from 52 patients at the University Hospital Leuven (UZ Leuven, Belgium) after written informed consent. The study was approved by the UZ/KU Leuven Ethical Committee prior to patient recruitment, and received the study number ML3452. The study was registered at clinicaltrials.gov under the number NCT00936104. After resection, tumor blocks were minced into small pieces and incubated with collagenase type IV (1 mg/ml in Medium 199; Invitrogen, Grand Island, NY) for 2–3 hr while mechanically dispersed at regular 20-min time points. The final cell suspension was filtered through a 40 µm nylon mesh (BD Biosciences, Erembodegem, Belgium), and cell number and viability determined. The viability of the cells obtained after dispersion of the PDAC tissues was routinely ∼50% and the yield of 400 mm^3^ freshly obtained PDAC samples was approximately 3×10^6^ living cells.

SP analysis was done as described before ^3^. Cells were incubated with Hoechst33342 (Sigma-Aldrich, Bornem, Belgium) during 90 min at a final concentration of 5 µg/ml, and analysed by FACS (FACSVantage SE, equipped with FACS DIVA software, version 6.0; BD Biosciences). The lasers used were: near-UV (375 nm, 10 mW output), blue (488 nm, 13 mW) and yellow-green (561 nm, 30 mW). The filters used were: 450/40 for Hoechst blue; 630/22 and 610 LP for Hoechst red; 530/30 and 520 LP for FITC; and 582/15 for PE. Propidium iodide (2 µg/ml; Sigma-Aldrich) was added to mark non-viable cells, excluding the debris in the Hoechst-blue and Hoechst-red channel. Dual-wavelength FACS analysis identified a side branch of ‘Hoechst-low’ cells as the SP, further verified by co-adding verapamil (100 µM; Sigma-Aldrich) which results in reduction of the SP size by blocking the dye efflux through multidrug transporter(s). The main population (MP) was gated as the bulk of ‘Hoechst-bright’ cells. For further characterization, PDAC cells were immunostained for the endothelial marker CD31 and the hematopoietic/immune marker CD45. After incubation with Hoechst, the cells were dissolved in staining buffer (PBS +2% FBS), and fluorescein (FITC)-labeled anti-human CD31 and phycoerythrin (PE)-labeled anti-human CD45 antibodies were added using dilutions recommended by the manufacturer (BD Biosciences). Both isotype (BD Biosciences) and positive controls were performed. Additional stainings were not done as these could impede analysis of the FACS outcome.

### Whole-genome Expression Analysis by Microarray

25000 SP and 25000 MP cells (total or depleted from CD45^+^/CD31^+^ cells) were sorted by FACS into cold lysis solution of the RNeasy Micro Kit (Qiagen, Venlo, The Netherlands). Total RNA was extracted according to the instructions of the manufacturer. RNA concentration and purity were determined spectrophotometrically using the Nanodrop ND-1000 system (Nanodrop Technologies, Wilmington, DE) and RNA integrity was assessed using a Bioanalyser 2100 (Agilent Technologies, Santa Clara, CA). Only samples with RNA Integrity Number (RIN) ≥7.5 were used for further microarray analysis at the VIB Nucleomics Core (www.nucleomics.be). RNA was amplified with the NuGEN Pico WTA kit (Nugen Technologies, Santa Carlos, CA) and subsequently biotinylated. The final aRNA mixture was purified and fragmented, and then hybridized on Affymetrix HG U133 Plus 2.0 arrays (Affymetrix, Santa Clara, CA), followed by staining and washing in a GeneChip fluidics station 450 (Affymetrix) according to the manufacturer’s procedures. To measure the raw probe signal intensities, chips were scanned using a GeneChip scanner 3000 (Affymetrix) and analysed with the affy_1.26.0 package (Bioconductor, Seattle, WA).

### Statistical and Functional Analysis of Microarray Data

The raw microarray data were normalized within arrays and between arrays following the Robust Multichip Average (RMA) procedure [13]. The MAS 5.0 algorithm (Microarray suite user guide, version 5; Affymetrix 2001) was used to assess detection above background. From the 54616 probe sets, 10255 were below background and omitted from further analysis. Principal Component Analysis (PCA) was done with the pcurve package from Bioconductor and used to compute the main sources of variation between the samples. For comparative analysis between SP and MP, data were paired per patient. The limma package from Bioconductor was used to assess the contrast between SP and MP [14]. Statistical significance of this contrast was tested with a moderated t-test (implemented in limma). Significantly up- or downregulated genes were defined as genes with ≥2-fold change (log_2_ SP/MP≥1 or ≤−1), in combination with p<0.001. Classical schemes to adjust for multiple testing can result in low statistical power for microarray studies. The stringent cut-off of p<0.001 was used as an alternative, pragmatic approach to balance the number of false positives and false negatives [15]. Gene expression data are available from the Gene Expression Omnibus (GEO, http://www.ncbi.nlm.nih.gov/projects/geo/) through series accession number GSE42404. Functional pathway analysis on all significantly differentially expressed probe sets was done with the Ingenuity Pathway Analysis (IPA) program (Ingenuity Systems, www.ingenuity.com; Redwood City, CA), built on the Ingenuity Knowledge Base, a manually curated literature database. All probe sets with a corrected p-value <0.001 and a fold change of >2 or <−2 were used as input. In case multiple probes referred to the same gene, the average log_2_-ratio of the set of entry values for this gene was taken for further analysis. Generated networks were ordered by a score meaning significance, estimated as the ratio of the number of input probes that mapped to the pathway divided by the total number of pathway probes. Significance of biological functions and canonical pathways were tested by the Fisher’s exact test p-value after application of Benjamini-Hochberg method of multiple testing correction. Pathways were considered significant when p<0.05. Additional identification of biological functions (Gene Ontology, GO) and KEGG (Kyoto Encyclopedia of Genes and Genomes) pathways was performed using The Database for Annotation, Visualization and Integrated Discovery (DAVID, version 6.7, http://www.david.abcc.ncifcrf.org). For this analysis, probe sets with a corrected p-value <0.001 and a >2.0-fold change were used as input. The EASE score, a modified Fisher’s exact p-value test, was used to adjust for multiple testing.

### Development of a Gene Signature

A supervised learning strategy was applied to the microarray data of the CD45^−/^CD31^−^ SP and MP populations (i.e. the purified SP or pSP, and the pMP). A list of 200 genes of interest was composed based on a thorough literature study using the following terms: ‘pancreatic cancer’, ‘cancer stem cell’, ‘side population’, ‘canonical pathways’, ‘stemness genes’, ‘epithelial mesenchymal transition (EMT)’, ‘multidrug resistance’, ‘oncogenes’ and ‘polycomb genes’. The preprocessed data were limited to 512 probe sets (195 genes) that had gene symbols in the list of 200 genes of interest. In total, 29 different classification approaches were used, representing various combinations of 5 classification models, 3 modes of feature selection and 3 ranking methods [16]. Evaluation of these approaches was done using the leave-one-out-cross-validation (LOOCV) to create a receiver operating characteristic (ROC) curve [17]. The univariate feature selection methods turned out to have no added value since the optimal approach with highest area under the ROC curve (AUC) and lowest balanced error rate (BER) consisted of a support vector machine without prior feature ranking or selection (data not shown). To further reduce the gene signature, we used the PINTA strategy for gene candidate prioritizing [18]. By looking at differential expression of the neighbourhood of a gene in a genome-wide protein-protein interaction network, the PINTA strategy effectively performs multivariate feature selection through the incorporation of prior knowledge of molecular biology. A group of 32 genes was assigned as being significant, and the probe sets with the highest expression level were chosen. Finally, the PINTA signature was further narrowed to those 10 genes with the largest fold change (up or down).

The predictive value of the 32-gene and the reduced 10-gene signature was validated with LOOCV on different SP *versus* MP data sets.

### nCounter Analysis

Between 2006 and 2010, tissue samples were collected, after informed consent, from patients who underwent pancreatic resection for PDAC. Samples were stored at −80°C in RNALater (Qiagen). From the primary tumor of 143 patients and from surrounding non-tumoral pancreatic (control) tissue of 14 patients, total RNA was extracted using the RNeasy Mini kit (Qiagen) according the manufacturer’s instructions. Only samples with an RNA integrity number (RIN) of >7.0 were used for further analysis, i.e. 78 PDAC samples (male/female ratio: 41/37; age: 32–80 yr with median of 64 yr) and 6 control tissues (male/female ratio: 4/2; age: 51–66 yr with median of 63 yr). Tumor characteristics and prognostic features (with a follow-up until July 2011) of the 78 patients are provided (see Table S1). Using the nCounter system (Nanostring Technologies, Seattle, WA), expression levels of the above-defined signature genes were quantified in pancreatic tumor *versus* surrounding tissue (VIB Nucleomics Core) [19].

### ABCB1 Immunohistochemistry in PDAC Resection Specimens

To analyze the expression of ABCB1 protein by immunohistochemistry, 5-µm sections were prepared from formalin-fixed paraffin-embedded PDAC specimens of the 11 patients whose pSP and pMP RNA were used for microarray analysis. After deparaffinization and rehydration of the slides, target retrieval was done with EnVision FLEX Target Retrieval Solution (Dako, Glostrup, Denmark) during 20 min. Endogenous peroxidase activity was blocked using EnVision Peroxidase-Blocking Reagent (Dako) for 5 min. Sections were incubated with primary antibody (anti-human ABCB1 from Monosan, Uden, The Netherlands) at the recommended dilution (1/20) for 30 min at room temperature. Subsequently, the slides were processed using the EnVision Dual Link (Dako). The complex was visualized with DAB (3,3′-diaminobenzidine; Dako), followed by a hematoxylin (Dako) counterstaining. Pictures were taken with a Leica DC 300 camera on a Leica DMLB microscope.

### Statistical Analysis

SAS Statistical Software (version 9.1; SAS Institute, Cary, NC) was used for all statistical analyses. A p-value <0.05 was considered statistically significant. Disease-free survival (DFS) and overall survival (OS) were calculated using the Kaplan-Meier method (Table S1). To test prognostic value of genes, univariate analysis for DFS and OS was performed using Cox regression with and without restricted cubic splines (RCS), followed by multivariate analysis for OS using Cox regression. A selected model was used for analysis independent of variables already correlated with survival (ECLNI, gender, pM and pT; see Table S1).

## Results

### Human Pancreatic Cancer Contains a Side Population

**Figure 1:**
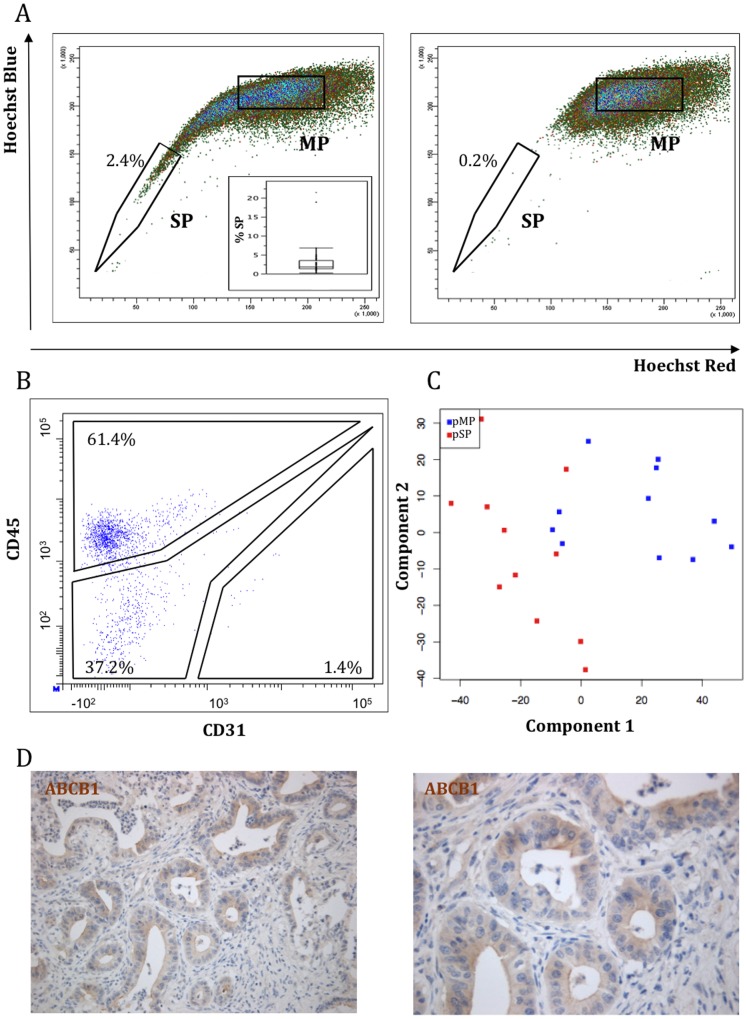
Identification and molecular characterization of the human PDAC SP. A) Dot plot of dual-wavelength FACS analysis of fresh human PDAC cells after incubation with Hoechst33342 depicting a tail of ‘Hoechst-low’ cells (SP), relative to a larger bulk of ‘Hoechst-bright’ cells, the main population (MP) (*left panel*). A representative example is shown and the SP proportion indicated. PI^pos^ (dead) cells were excluded from analysis for Hoechst33342, CD45 and CD31 labelling. The boxplot (*inset*) summarizes the SP proportions of the 52 PDAC samples analyzed. Verapamil blocks Hoechst efflux, thereby reducing the SP size and confirming the SP phenotype (*right panel*). B) FACS dot plot of the PDAC SP, immunostained for CD45 and CD31. A representative example is shown. Numbers indicate the proportions of CD45^+^, CD31^+^ and CD45^−/^CD31^−^ cells within the SP. C) Principal Component Analysis (PCA) of whole genome expression profiles obtained from CD45^−/^CD31^−^ SP (pSP, *red*) and CD45^−/^CD31^−^ MP (pMP, *blue*) (n = 11). D) Immunohistochemical staining of ABCB1 in PDAC resection samples (n = 11). A representative example (4% SP in FACS analysis) is shown. Original magnification: x200 (*left*) and x400 (*right*).

Human PDAC resection specimens were analyzed for the presence of a side population (SP). In all samples examined (n = 52), a SP was identified representing between 0.2 and 21.5% (median: 1.8%) of the total PDAC cells (Fig. 1A).

SP and ‘main population’ (MP) cells (see Fig. 1A for gating) were sorted by FACS and subjected to whole-genome expression profiling (n = 10 PDAC). Ingenuity Pathway Analysis (IPA) revealed in the SP genes and pathways related to immunological processes, such as ‘T-helper cell differentiation’, ‘Communication between innate and adaptive immune cells’ and ‘Dendritic cell maturation’ (data not shown). Immune-type cells, as well as endothelial cells, are known to co-segregate at varying extents in the SP of tissues and tumors. Therefore, we analyzed the human PDAC SP by flow cytometry for the expression of the immune/hematopoietic marker CD45 and the endothelial marker CD31. About 60% of the SP cells are CD45^+^ (median: 61.4%, range: 19.5–75.4%; n = 18) while only a small number of SP cells are CD31^+^ (median: 0.7%, range: 0.2–2.1%; n = 15) (Fig. 1B). CD45^+^ and CD31^+^ cells were also observed in the MP at comparable levels (median 63.7% and 1.6%; and range: 41.1–75.4% and 0.1–6.7%, respectively). For subsequent analyses, the SP and MP were depleted from the CD45^+^ and CD31^+^ cells.

### The Purified PDAC SP Shows Expression of CSC-associated Genes

We sorted the CD45^−/^CD31^−^ SP and CD45^−/^CD31^−^ MP cells (further referred to as purified SP or pSP, and purified MP or pMP, respectively) from 11 PDAC samples and again performed whole-genome expression profiling. Principal Component Analysis (PCA) shows that the pSPs cluster and segregate from the pMPs (Fig. 1C).

Of the 1861 probe sets that are differentially expressed (≥2-fold, p<0.001), 993 are upregulated in the pSP and 868 are downregulated (see GEO, accession number GSE42404). The multidrug transporter *ABCB1* (*MDR1*, *P-glycoprotein*) is highly upregulated in the pSP *versus* the pMP (fold: 5.3, p<0.001), and is potentially underlying the SP phenotype. Immunohistochemical analysis confirmed ABCB1 expression in PDAC tumor cells, mainly located at the apical surface of the cells (n = 11 PDAC samples; Fig. 1D). *ABCG2*, another transporter that can mediate SP efflux, is not differentially expressed (p>0.5), but the microarray signal was generally low and not above background in half of the samples. Interestingly, previously described pancreatic CSC markers (including *CD44, CD133* and *CXCR4*) are significantly overexpressed in the pSP (Table S2). In addition, other (cancer) ‘stemness’ genes (like *LGR4, SOX9, KLF5* and *MET*) are also upregulated in the pSP.

Of all differentially expressed probe sets, 1690 could be mapped to genes in the Ingenuity Knowledge Base and were subjected to IPA (Table S3). The networks most upregulated in the pSP are ‘Amino acid metabolism’ (which includes *HNF4A* or ‘Hepatocyte nuclear factor 4 alpha’, fold: 2.7, p<0.001), ‘Cell-to-cell signaling’ (which includes *TJP2* or ‘Tight junction protein 2′, fold: 2.4, p<0.001) and ‘Cellular growth and proliferation’ (which includes *EPHA2* or ‘Ephrin receptor A2’, fold: 2.1, p<0.001; and *EPHA4*, fold: 2.6, p<0.001).

Biological functions enriched in the pSP *versus* the pMP encompass ‘Cellular movement’, ‘Cell-to-cell signaling and interaction’, ‘Cellular growth and proliferation’, ‘Embryonic development’, ‘Tumor morphology’ and ‘Cancer’ (Table S3). To discriminate between pSP- and pMP-associated biological functions in more detail, DAVID analysis was applied. Clusters related to ‘Cell-cell junction’ (Enrichment Score or ES: 6.8), ‘Protein kinase activity’ (ES: 3.4), ‘Development’ (ES: 3.0), ‘Mitogen activated (MAP) kinase activity’ (ES: 1.8), ‘Integrin signaling’ (ES: 1.6) and ‘Regulation of apoptosis’ (ES: 1.4) are enriched in the pSP.

Canonical pathways, based on differentially expressed genes, were also analyzed using IPA. A number of 46 canonical pathways reached the significant SP enrichment cut-off of p<0.05, including ‘Human embryonic stem cell pluripotency’, ‘Tight junction signaling’, ‘NF-κB signaling’, ‘Wnt/β-catenin signaling’, ‘Integrin signaling’ and ‘Ephrin signaling’ (Table S3). Further detailed analysis using DAVID (KEGG pathways) showed upregulation of the ‘ErbB signaling pathway’ (including *ErbB3*, fold: 2.1, p<0.001) in the pSP.

### Gene Signatures that Discriminate the PDAC pSP from the pMP

Using a supervised learning strategy applied to the whole-genome expression data from the 11 CD45^−/^CD31^−^ pSP and pMP pairs as analyzed above, we developed a set of 512 probe sets (195 genes) as potential ‘pSP gene signature’. The accuracy to discriminate between pSP and pMP using this signature was 95% in the training cohort, as evaluated with LOOCV (see Materials and Methods).

Using PINTA (see Materials and Methods), this large pSP gene signature was reduced to 32 genes, of which 19 are upregulated and 13 downregulated (Table S4). Validation of this reduced pSP gene signature in the original dataset (n = 11 PDAC samples) demonstrated a 91% classification accuracy of pSP and pMP, comparable to the prediction using the 195-gene signature. Within the 32-gene signature, genes upregulated in the pSP include previously proposed pancreatic CSC markers (*CXCR4*, *CD133*) or play a role in multidrug resistance (*ABCB1*) and in pathways important in tumorigenesis and tumor progression (see Table S4).

Finally, we further narrowed the gene signature to those 10 genes with largest fold up- or downregulation (Table S4, in bold). The accuracy of this condensed gene signature to distinguish pSP from pMP (90%) is comparable to the discriminatory power of the 32-gene set.

As an ultimate test of the discriminating value of the 32- and 10-gene signatures, we applied both sets to an additional, independent series of 10 SP/MP pairs using LOOCV. Both gene signatures presented a discriminating accuracy between SP and MP of 85%.

### Prognostic Value of ABCB1 and CXCR4

To investigate whether genes of the pSP (32- and 10-)gene signatures have prognostic value, expression levels were first determined in an independent series of patients (n = 78; Table S1) in tumor *versus* surrounding tissue using nCounter analysis (see Materials and Methods) and then subjected to univariate analysis for potential correlation with prognosis/survival. First, signature genes overexpressed in the pSP are related to prognosis whereas no correlation was found with signature genes downregulated in the pSP (data not shown). Moreover, upregulation of *ABCB1* and *CXCR4* is significantly correlated with worse prognosis, i.e. with lowest OS and DFS (Table S5). Multivariate analysis was then performed on the genes with p<0.1 in univariate analysis (*ABCB1*, *ADAM10*, *CDH1*, *CXCR4*, *ESRP1*, *MMP1*, *RAB25*, *ST14*), designating *ABCB1* as an independent predictor of poor OS (Table S5).

## Discussion

In the present study, we identified a SP in human pancreatic cancer. To our knowledge, this is the first report describing the presence of a SP in PDAC analyzed from patients. Like in other tissues and tumors, the PDAC SP contains a fraction of CD31^+^ endothelial cells and CD45^+^ immune cells [20]. In the current study, we focused on the non-endothelial, non-immune SP (pSP) and scrutinized its gene expression profile. The pSP mainly represents tumoral epithelial cells given the high expression of *CDH1*, *EPCAM, TJP1* and *TJP2* [21]. In this PDAC pSP we found upregulation of the multidrug transporter *ABCB1*, which may be responsible for chemoresistance of these cells. Immunostaining confirmed the presence of ABCB1 in PDAC, mainly located at the apical surface of the tumor cells where active drug transport may occur. Upregulation of apoptosis-regulating factors (*Bcl2L11,* fold: 2.9, p<0.001; *EPHA2*, see Results) in the PDAC pSP may further support its chemoresistant character. We have recently shown that the SP is highly resistant to gemcitabine in a mouse model in which PDAC was grown as xenografts [12]. Resistance of the SP to gemcitabine has also been reported in pancreatic cancer cell lines *in vitro* [8], [9], [11]. Taken together, the SP appears to represent a chemoresistant subpopulation of the pancreatic tumor, and may thus be one of the culprits for treatment failure and cancer recurrence.

In several types of cancer, the SP is enriched for candidate CSC [7], [22]. We found that markers recently identified in putative CSC populations of human pancreatic cancer, are upregulated in the PDAC pSP. In addition, we detected some other ‘cancer stemness’ markers (e.g. *LGR4, SOX9, KLF5, MET*) as well as pancreatic embryogenesis-related factors which may be re-initiated in CSC (e.g. *HNF4A*) [5], [6], [23]–[26]. Moreover, *in silico* functional analysis of the expression data highlights the occurrence of pathways of embryonic development and embryonic stem cell pluripotency in the PDAC SP, further supporting its ‘stemness’. Also in pancreatic cancer cell lines, the SP appeared enriched in CSC [9]–[11]. Nevertheless, it is clear that more evidence is needed to conclusively demonstrate the CSC phenotype of the pSP, including tumorigenic activity *in vitro* (sphere formation) and *in vivo* (tumor growth in immunodeficient mice).

The PDAC pSP may represent an interesting therapeutic target given its chemoresistance- and CSC-associated character. The pSP cells express high levels of *CXCR4,* which upon activation, may stimulate the cells to move or to undergo ‘epithelial-mesenchymal transition’ (EMT), a process driving cancer progression and malignancy. In pancreatic cancer cell lines, the SP can indeed be activated towards EMT when treated with TGFβ [21]. In the PDAC pSP, we observed upregulation of *SMAD3*, known to mediate TGFβ signaling. Further interestingly, *CXCL12*, the ligand of CXCR4, is highly expressed in the surrounding pancreatic tissue of the PDAC tumor (data not shown) and may lure cells out of the tumor for invasion or further dissemination. In analogy, CXCR4 expression has been found to characterize a subpopulation of the CD133^+^ pancreatic CSC that seem to be reponsible for the metastasis of the tumor [6], [27].

Upregulation of *EPHA2* and *EPHA4* in the pSP is in line with an important role of the Ephrin signaling pathway in PDAC pathogenesis. Expression of EPHA2 in PDAC has been associated with increased invasive and metastatic ability and poor survival [28]. Knockdown of EPHA4 expression decreases PDAC cell viability [29]. In addition, we found that the ErbB signaling pathway is upregulated in the pSP (KEGG analysis), including the higher expression of *ErbB3*. The ErbB3 receptor plays a crucial role in pancreatic development as well as in pancreatic tumorigenesis. Overexpression of *ErbB3* correlates with advanced PDAC stage and decreased overall survival, and recently, its role as potent mediator of PI3K signaling has been highlighted [30], [31]. Moreover, activation of ErbB3 may be essential in the resistance to single-agent EGFR inhibition. Specifically inhibiting ErbB3 activity (e.g. with MM-121) appears promising *in vitro,* and thus may also have clinical potential [31]. Both the Ephrin and ErbB signaling pathways may present potential therapeutic targets in PDAC. It should be noted that our study did not examine the prognostic value of *EPHA2* and *ErbB3* since these genes were not included in the pSP signatures evaluated against survival.

The developed pSP gene signatures trustfully discriminate the pSP from the pMP. Some of the upregulated genes are related to CSC/’cancer stemness’ such as *CXCR4, CD133, EPCAM* and *SOX9* [5], [6], [24], while others are associated with apoptosis (*FASLG, TJP2, DUSP4*), the MAPK pathway (*MAP2K4, DUSP4*), and chemoresistance (*MMP1*, an *ETS1* target gene) [32], [33]. In addition, the signature contains genes related to increased motility and invasion (*ADAM10, RAB25, DUSP4, CXCR4*). Also some of these genes may represent promising targets.

Finally, we evaluated the prognostic relevance of genes included in the pSP signatures. *CXCR4* and *ABCB1* are negatively related to survival after curative resection, which is also found in other reports [34], [35]. CXCR4 has previously been connected with metastatic PDAC CSC and inhibiting CXCR4 with AMD3100 *in vivo* resulted in reduced metastasis [6], [27]. Our data support CXCR4 as an interesting pSP−/CSC-associated target, which additionally presents prognostic relevance. ABCB1 has been studied as a potential therapeutic target in other cancers. In a model for ovarian cancer, drug resistance was reverted after downregulation of ABCB1 by shRNA [36]. We have shown that ABCB1 may also play a role in PDAC, as it is related to therapy resistance and affects patient outcome [37], [38]. However, treatment-related toxicity needs substantial consideration, as ABCB1 is also present on a large variety of normal cells participating in physiological processes. Therefore, further study is needed to confirm the clinical impact of the SP. For instance, potentially important genes upregulated in the SP may be knocked down by siRNA/shRNA, to evaluate the impact on tumorigenesis in immunodeficient mice.

In conclusion, we identified a SP in human PDAC. Gene expression profiling revealed chemoresistance- and CSC-associated characteristics and yielded (new) candidate therapeutic targets with potential prognostic value.

## Supporting Information

Table S1 (DOCX)

Table S2 (DOCX)

Table S3 (DOCX)

Table S4 (DOCX)

Table S5 (DOCX)
