# Supplementary material for: Human Pancreatic Cancer Contains a Side Population Expressing Cancer Stem Cell-Associated and Prognostic Genes
Source: PLoS One. 2013 Sep 17;8(9):e73968. doi: 10.1371/journal.pone.0073968 (PMC3775803; doi:10.1371/journal.pone.0073968)
Supplement: Table S1 — (DOCX) [file pone.0073968.s001.docx]

**Table S1.** Clinicopathological parameters of patients included in prognostic analysis

| **Patient characteristics** | **Number** | **Percentage** | **p-value 0S** |
| --- | --- | --- | --- |
| OS | 18.7 mo (95% CI: 12.4-25.6 mo) |  |  |
| DFS | 10.0 mo (95% CI: 7.4-12.4 mo) |  |  |
| Gender |  |  | 0.0242 |
| Male | 41 | 53% |  |
| Female | 37 | 47% |  |
| pG |  |  | 0.0622 |
| pG1 | 6 | 8% |  |
| pG2 | 22 | 28% |  |
| pG3 | 50 | 70% |  |
| pT |  |  | 0.0069 |
| pT2 | 11 | 14% |  |
| pT3 | 63 | 81% |  |
| pT4 | 4 | 5% |  |
| pN |  |  | 0.3454 |
| pN0 | 29 | 37% |  |
| pN1 | 49 | 83% |  |
| pM |  |  | 0.0134 |
| pM0 | 71 | 91% |  |
| pM1 | 7 | 9% |  |
| pR |  |  | 0.5664 |
| pR0 | 63 | 81% |  |
| pR1 | 15 | 19% |  |
| PNI |  |  | 0.0905 |
| No PNI | 10 | 13% |  |
| PNI | 67 | 87% |  |
| VI |  |  | 0.5695 |
| No VI | 25 | 32% |  |
| VI | 53 | 68% |  |
| LVI |  |  | 0.7234 |
| No LVI | 23 | 29% |  |
| LVI | 55 | 71% |  |
| ECLNI |  |  | 0.0114 |
| ECLNI | 25 | 34% |  |
| No ECLNI | 49 | 66% |  |

OS: overall survival; DFS: disease-free survival; mo: months; pG: pathological tumour grade; pT: pathological tumour size; pN: pathological lymph node status; pM: pathological metastasis; pR: pathological resection margin; PNI: perineural invasion; VI: vascular invasion; LVI: lymphovascular invasion; ECLNI: extracapsular lymph node invasion.
