# Supplementary material for: Human Pancreatic Cancer Contains a Side Population Expressing Cancer Stem Cell-Associated and Prognostic Genes
Source: PLoS One. 2013 Sep 17;8(9):e73968. doi: 10.1371/journal.pone.0073968 (PMC3775803; doi:10.1371/journal.pone.0073968)
Supplement: Table S2 — (DOCX) [file pone.0073968.s002.docx]

**Table S2**. Expression of stemness- or CSC-associated genes in the PDAC pSP *versus* pMP

|  | Gene symbol | Fold pSP/pMP | p-value |
| --- | --- | --- | --- |
| Previously identified pancreatic CSC markers | CD44 ^5^  EPCAM ^5^  CD24 ^5^  CD133 ^6^  CXCR4 ^6^ | 2.19  2.81  2.85  3.21  4.14 | 0.00018  0.00045  0.00027  0.00055  0.00139 |
| Other (cancer) ‘stemness’ markers | LGR4 ^23^  SOX9 ^21^  KLF5 ^22^  MET ^24^ | 2.14  2.40  3.26  4.86 | 0.00031  0.00016  3.66^E^06  0.00001 |
