# Supplementary material for: Human Pancreatic Cancer Contains a Side Population Expressing Cancer Stem Cell-Associated and Prognostic Genes
Source: PLoS One. 2013 Sep 17;8(9):e73968. doi: 10.1371/journal.pone.0073968 (PMC3775803; doi:10.1371/journal.pone.0073968)
Supplement: Table S3 — (DOCX) [file pone.0073968.s003.docx]

**Table S3.** Ingenuity Pathway Analysis (IPA) performed on PDAC pSP *versus* pMP

| IPA^a^ | | Selected genes^b^ |
| --- | --- | --- |
| **Networks** | Amino acid metabolism | **HNF4A, TM4SG4, TRMT6, MREG, SOX9** |
|  | Cell-to-cell signaling and interaction | **BCL10, E2F7, EPCAM, TJP1, TJP2** |
|  | Cancer | **CDH1, DKK1, DSP, NEDD9,** LEF1, TCF4, TGFB1I1 |
|  | Cellular growth and proliferation | **EPHA2, EPHA4, MAPKSP1, MST4, SERPINB1** |
|  | Embryonic development | **AGFG1, ARHGEF5, GOT1, GFI1, TGFA, TM4SF1, ETS2, CRB** |
| **Biological functions** |  |  |
| Diseases and disorders | Cancer | **ABCB1, AREG, CDH1, CD24, CD44**, CXCL12**, DKK1, FOXA2**, LEF1, **MET**, MMP2, **CD133**, TCF4, TWIST1 |
|  | Genetic disorder | **ABCB1, BCL2L11**, PLA2G2A, **FOXA1, TJP1**, LEF1, **CD133, SMAD3** |
|  | Gastro-intestinal disease | **ABCB1, CEACAM1, EPCAM**, FN1, **KRAS**, MSX1**, CD133, ST14**, **SMAD3** |
| Molecular and cellular functions | Cellular movement | AKT3, **CD24, CD44, CDH1, CEACAM1**, CXCL12, **DKK1**, DKK3, FN1, **MET**, LEF1, **SMAD3**, **SYK,** TGFB3, TWIST1, VIM |
|  | Cell-to-cell signaling and interaction | ADAM12, **CD24, CD44, CDH1**, FGFR1, FN1, **FOXA2**, MMP2, SERPINE1, **SOX9, MET** |
|  | Cellular growth and proliferation | **ABCB1, DKK1**, DKK3, **EPCAM**, FGFR1, LEF1, LOX, **SOX9, SYK**, TCF4, TWIST1, VIM |
| Physiological system development and functions | Tissue development | MSX1, PDGFRB, **MET, CDH1**, FN1, **EPHA4,** TGFBI, **TJP1,CD44, EPCAM, BMP2, HDAC9,** TGFB1I1, **SOX9, DKK1** |
|  | Embryonic development | SFRP2, FN1, NOG, **EFNB2, SOX9**, IGF1, **EDN1**, SERPINE1, TIMP2, PTX3, **ITGB1**, MMP2, **CEACAM1**, TGFB3 |
|  | Tumour morphology | FN1**, KRAS,** SERPINE1, FGFR1, VIM, MMP2**, CEACAM1, CDH1, SYK**, CXCL12, **CD44**, TGFB3, SFRP1, **EPHA2** |
| **Canonical pathways** | Tight Junction signaling | **TJP1, TJP2**, ACTA2, **CLDN1**, TGFB3, AKT3, ACTG2 |
|  | Human embryonic stem cell pluripotency | **TCF4, BMP2, SMAD3,** FGFR1, FZD1, NOG, PDGFRA, TGFB3, LEF1 |
|  | NF-kB signaling | **BMP2, KRAS, BCL10,** PDGFRA, PDGFRB |
|  | Wnt/β-catenin signaling | **TCF4,** SFRP2, FZD1, SOX11, **SOX9,** CDH2, **CDH1**, DKK3, **CD44**, TGFB3, AKT3, LEF1, **FZD5,** SFRP1, **DKK1** |
|  | HIF1α signaling | **KRAS,** MMP2, AKT3, **PIK3CB**, MMP11, MMP19 |
|  | Integrin signaling | **ITGB1**, ACTA2, **ITGA2, ITGA6**, **KRAS,** **BRAF, ITGAM**, ITGA9, ITGA11, AKT3 |
|  | FGF signaling | **MET,** FGFR1, FGF14, **MAP3K**1, AKT3, FGF11, STAT3, FGF7 |
|  | Ephrin signaling | **ITGB1,** **RAC2**, **ITGA2, EPHA4**, **KRAS**, STAT3, **EFNA1, EFNB2**, AKT3, PDGFD, **EPHA2** |

^a^IPA was performed on the 1690 differentially expressed probe sets (with entry in the Ingenuity Knowledge Base; [www.ingenuity.com](http://www.ingenuity.com)). The most significant networks, biological functions and canonical pathways are listed.

^b^A selection of genes contributing to the networks, biological functions or pathways, is given. *Bold*, upregulated in the pSP.
