# Supplementary material for: Human Pancreatic Cancer Contains a Side Population Expressing Cancer Stem Cell-Associated and Prognostic Genes
Source: PLoS One. 2013 Sep 17;8(9):e73968. doi: 10.1371/journal.pone.0073968 (PMC3775803; doi:10.1371/journal.pone.0073968)
Supplement: Table S4 — (DOCX) [file pone.0073968.s004.docx]

**Table S4**. Gene signatures discriminating the PDAC pSP from the pMP

| **Probe ID** | **Gene symbol^a^** | **Gene name** | **Fold pSP/ pMP** | **p-value** | **Pathway/ function** |
| --- | --- | --- | --- | --- | --- |
| 209993_at | **ABCB1** | ATP-binding cassette, sub-family B (MDR/TAP), member 1 | 5.27 | 2.78^E^-07 | Multidrug resistance,  SP phenotype |
| 211919_s_at | **CXCR4** | Chemokine (C-X-C motif) receptor 4 | 4.35 | 0.00222 | Chemotaxis, metastasis,  CSC marker |
| 225846_at | **ESRP1** | Epithelial splicing regulatory protein 1 | 4.34 | 0.00028 | Cancer progression, EMT |
| 201130_s_at | **CDH1** | Cadherin 1, type 1, E-cadherin (epithelial) | 3.56 | 0.00055 | WNT/β-catenin pathway,  epithelial marker |
| 204304_s_at | CD133 (PROM1) | Prominin 1 | 3.21 | 0.00055 | CSC marker |
| 216905_s_at | ST14 | Suppression of tumorigenicity 14 (colon carcinoma) | 3.10 | 0.00060 | Cancer invasion, metastasis |
| 226068_at | SYK | Spleen tyrosine kinase | 2.89 | 0.00002 | EMT,  link with integrins |
| 201839_s_at | EPCAM | Epithelial cell adhesion molecule | 2.81 | 0.00045 | CSC marker |
| 1405_i_at | CCL5 | Chemokine (C-C motif) ligand 5 | 2.79 | 0.05095 | Chemotaxis (immune cells) |
| 202936_s_at | SOX9 | SRY (sex determining region Y)-box 9 | 2.40 | 0.00016 | (Cancer) ‘stemness’ marker |
| 237132_at | TJP2 | Tight junction protein 2 (zona occludens 2) | 2.37 | 0.01059 | Intercellular interactions |
| 205396_at | SMAD3 | SMAD family member 3 | 2.35 | 0.01077 | TGFβ pathway |
| 206118_at | STAT4 | Signal transducer and activator of transcription 4 | 2.30 | 0.00183 | TGFβ pathway |
| 214895_s_at | ADAM10 | ADAM metalloproteinase domain 10 | 1.93 | 0.00076 | Notch pathway,  TNFα |
| 204015_s_at | DUSP4 | Dual specificity phosphatase 4 | 1.87 | 0.04654 | MAPK pathway |
| 203266_s_at | MAP2K4 | Mitogen-activated protein kinase kinase 4 | 1.74 | 0.00076 | MAPK pathway |
| 204475_at | MMP1 | Matrix metalloproteinase 1 (interstitial collagenase) | 1.71 | 0.04654 | Degradation of extracellular matrix |
| 210865_at | FASLG | Fas ligand | 1.63 | 0.02925 | Apoptosis |
| 218186_at | RAB25 | RAB25, member RAS oncogene family | 1.57 | 0.00852 | Tumor development and aggressiveness,  cancer cell motility |
| 203868_s_at | VCAM1 | Vascular cell adhesion molecule 1 | -1.67 | 0.03835 | Adhesion to endothelial cells |
| 220983_s_at | SPRY4 | Sprouty homolog 4 (Drosophila) | -1.99 | 0.00679 | MAPK and FGF pathway inhibitor |
| 212764_at | ZEB1 | Zinc finger E-box binding homeobox 1 | -2.10 | 0.00270 | EMT |
| 221558_s_at | LEF1 | Lymphoid enhancer-binding factor 1 | -2.46 | 0.00030 | WNT/β-catenin pathway |
| 203878_s_at | MMP11 | Matrix metalloproteinase 11 (stromelysin 3) | -2.76 | 0.00034 | Degradation of extracellular matrix |
| 213943_at | TWIST1 | Twist homolog 1 | -2.94 | 0.00044 | EMT |
| 231798_at | NOG | Noggin | -3.31 | 0.00084 | BMP inhibitor,  TGFβ pathway |
| 213891_s_at | **TCF4** | Transcription factor 4 | -3.53 | 0.00029 | WNT/β-catenin pathway inhibitor |
| 209651_at | **TGFB1I1** | Transforming growth factor beta 1 induced transcript 1 | -4.12 | 0.00027 | TGFβ pathway |
| 211161_s_at | **COL3A1** | Collagen, type III, alpha 1 | -4.30 | 0.00009 | Stromal component |
| 209687_at | **CXCL12** | Chemokine (C-X-C motif) ligand 12 | -5.37 | 3.82^E^-07 | Ligand of CXCR4 |
| 203649_s_at | **PLA2G2A** | Phospholipase A2, group IIA (platelets, synovial fluid) | -6.27 | 1.54^E^-06 | WNT/ β-catenin/TCF target |
| 215446_s_at | **LOX** | Lysyl oxidase | -6.33 | 8.55^E^-08 | EMT |

**^a^**The genes with largest fold difference (bold) were selected for the 10-gene signature.
