# Supplementary material for: Human Pancreatic Cancer Contains a Side Population Expressing Cancer Stem Cell-Associated and Prognostic Genes
Source: PLoS One. 2013 Sep 17;8(9):e73968. doi: 10.1371/journal.pone.0073968 (PMC3775803; doi:10.1371/journal.pone.0073968)
Supplement: Table S5 — (DOCX) [file pone.0073968.s005.docx]

**Table S5.** Prognostic value of SP signature genes

| **Gene name** | **Univariate analysis** | | | **Multivariate analysis** | |
| --- | --- | --- | --- | --- | --- |
|  | HR (95% CI) | p-value | p-value RCS | HR (95% CI) | p-value |
| **ABCB1** | 1.23 (0.96-1.58) | 0.0945 | 0.0101 | 1.40 (1.04-1.89) | 0.0266 |
| **CXCR4** | 1.24 (0.88-1.76) | 0.2136 | 0.0121 | 1.10 (0.68-1.79) | 0.6934 |

HR: hazard ratio; CI: confidence interval; RCS: a restricted cubic spline represents the HR
